# William Cary Barrett

**Published:** 1903-10

**Authors:** 


### WILLIAM CARY BARRETT.

UPON another page will be found a sketch of the life of Dr. Barrett, whose death in Germany on August 22, while not unexpected, brought with it a feeling of deep personal, as well as professional, loss.

The place which Dr. Barrett will hold in the dental profession must be left for time to determine. It may not be possible for his contemporaries to deal justly with such a character. He was the embodiment of energy, and this activity, mental and physical, naturally led him into antagonistic relations with many, while, at the same time, it drew a very large number of active and influential men into his life circle.

The position which he occupied was a unique one in his profession. Well grounded in its foundation principles, he seemed a law unto himself, leading him to differ with his colleagues upon many points of practice, and yet withal maintaining a position in general accord with recognized procedures. This trait in his character is very markedly illustrated in his work on "Oral Pathology and Practice."

That he was often misjudged by those brought into intimate relations with him was due largely to his peculiar temperament that could not take kindly to opposition, and this frequently created antagonisms that in his cooler and better moments he regretted. It is not surprising, therefore, that his motives were frequently misinterpreted. Underlying his apparently aggressive nature was, unquestionably, a sincere devotion to the best interests of his profession. He labored, through his own methods, that dentistry thereby might be aided to advance to a higher standard during his life, and that his efforts were not lost in his generation is manifest in the general uplifting of this calling during the years he was devoted to its interests. While he had many coworkers during the last half of the nineteenth century who were equally zealous in advancing professional standards, very few, perhaps none, threw themselves so unreservedly and with a more self-sacrificing spirit, as he did, into the whirl of professional activities. As an editor he was always forcible and frequently aggressive, and he left no room for doubt upon the minds of his readers as to his meaning.

His labors in the educational field were more productive of results than perhaps in any other direction to which his energies were extended. His force of character made a way where a less active and courageous mind would have faltered. He was always prepared for any advance that would make his profession more worthy of respect, hence in the National Association of Dental Faculties he never feared results where more timid men hesitated. This was peculiarly apparent when, as chairman of the Foreign Relations Committee, he attacked, almost single-handed, the dealers in diplomas in Chicago, and exposed, as never before, this nefarious traffic. It was a courageous effort, and, while eventually aided by others, the real credit for success attained in crushing out this fraudulent work must be given to him. It is questionable whether any other man in dentistry, or out of it, would have sacrificed so much with no hope of ultimate reward beyond the consciousness of work well done.

In whatever light Dr. Barrett be viewed, the conclusion must be that he was one of the remarkable men of his century, and, had he adopted a broader field for his life-work, would have made a world-wide reputation. For this self-sacrifice he should be remembered with gratitude, for he preferred to aid a struggling profession rather than seek honors where honors are more readily earned.

The permanent place that Dr. Barrett should fill in contemporary or future thought cannot be considered here. Complex characters, such as he possessed, cannot be judged properly in their lifetime. His errors in judging others and misconstruing motives of action may be allowed to lapse into forgetfulness, but his energy, his work bravely performed as he understood it, will live and serve as an encouragement to the faint-hearted to move forward in the pathway that leads directly to the highest, allowing no discouragements to block the way to that standard that means a truly professional life.

